# Supplementary material for: Benchmarking imputation methods for network inference using a novel method of synthetic scRNA-seq data generation
Source: BMC Bioinformatics. 2022 Jun 17;23:236. doi: 10.1186/s12859-022-04778-9 (PMC9204969; doi:10.1186/s12859-022-04778-9)
Supplement: Supplementary file 1 — Additional file 1. Additional figures demonstrating typical output of Biomodelling, the impact of varying the sparsity parameter, gene-gene correlations, inference results of 20 and 50 gene cases and the impact of varying the number of combination reactions. [file 12859_2022_4778_MOESM1_ESM.pdf]

Supplemental material for "Benchmarking imputation methods for network inference using a novel method of synthetic scRNA-seq data generation"

1 Example output from synthetic scRNA-seq data generation method

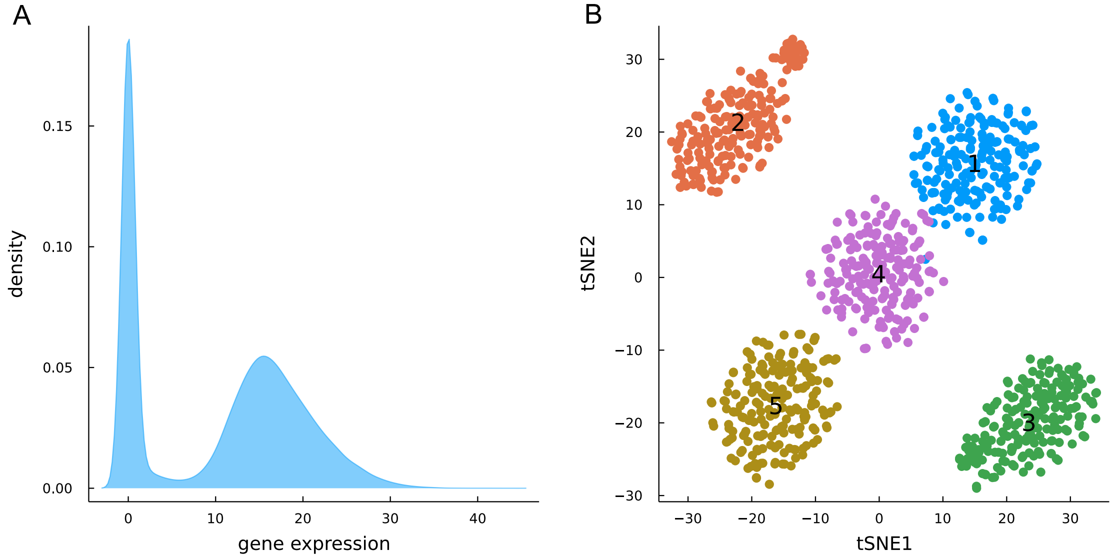

Supplemental Figure 1: Typical output of our synthetic scRNA-seq data generation method. (A) Kernel density plot of typical 50 gene network with sparsity equal to 0.04. (B) Low dimensional visualisation of typical synthetic data using tSNE method. To generate this plot, we combined data from 5 different 50 gene networks and used tSNE with perplexity parameter set to 20.

2 Choosing threshold parameter for network inference algorithms

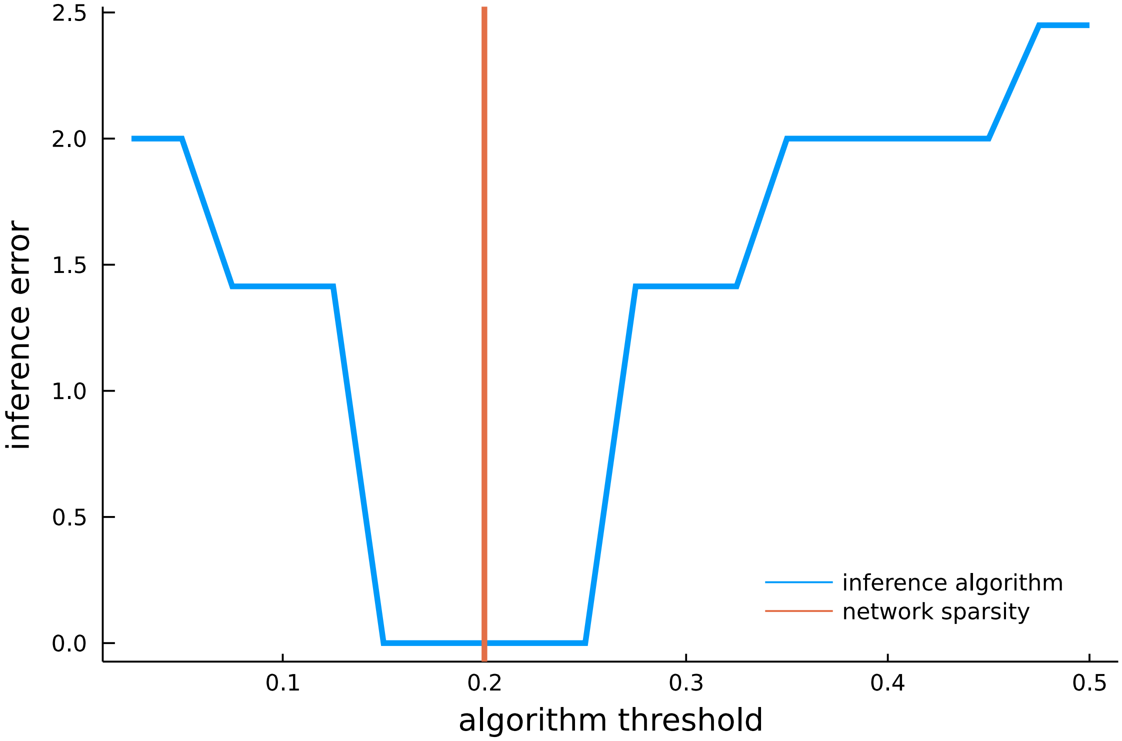

Supplemental Figure 2: Impact of varying threshold parameter for network inference algorithms: plot showing PIDC inference error (as defined by the l_2 norm of predicted adjacency matrix with ground truth adjacency matrix) as a function of the algorithm threshold for a 5-gene network toy example. The orange solid line is the network sparsity and the blue solid line represents the network inference algorithm error.

3 Example gene-gene correlations

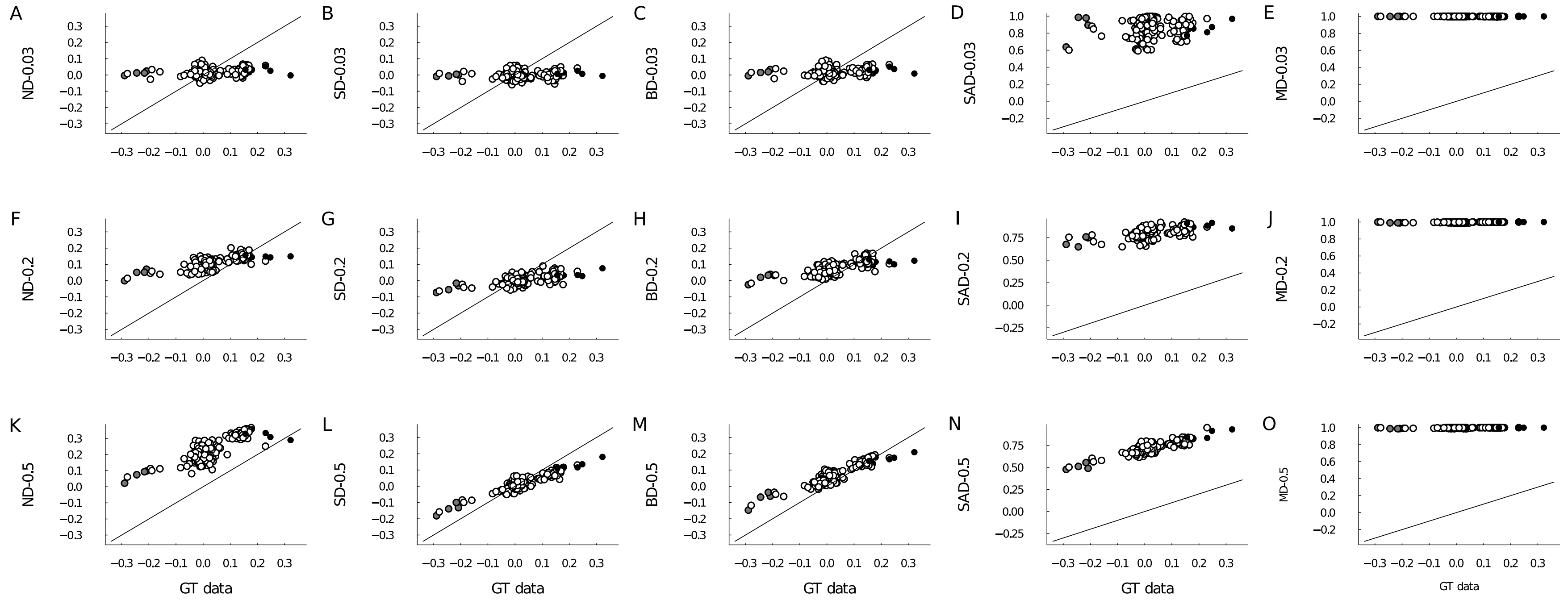

Supplemental Figure 3: Impact of imputation on Pearson gene-gene correlations : results for capture efficiency rates 0.03, 0.2 and 0.5 are shown in the first, second and third row respectively and the imputation/scaling methods appear in the y-axis label of each plot. The x-axis show the gene-gene correlations in GT data. The black solid line represents the linear equation $x = y$, black dots are activation type reactions, grey dots are inhibition type reactions and open circles represent non-reacting type.

4 Inference precision: 20 gene additive regulation

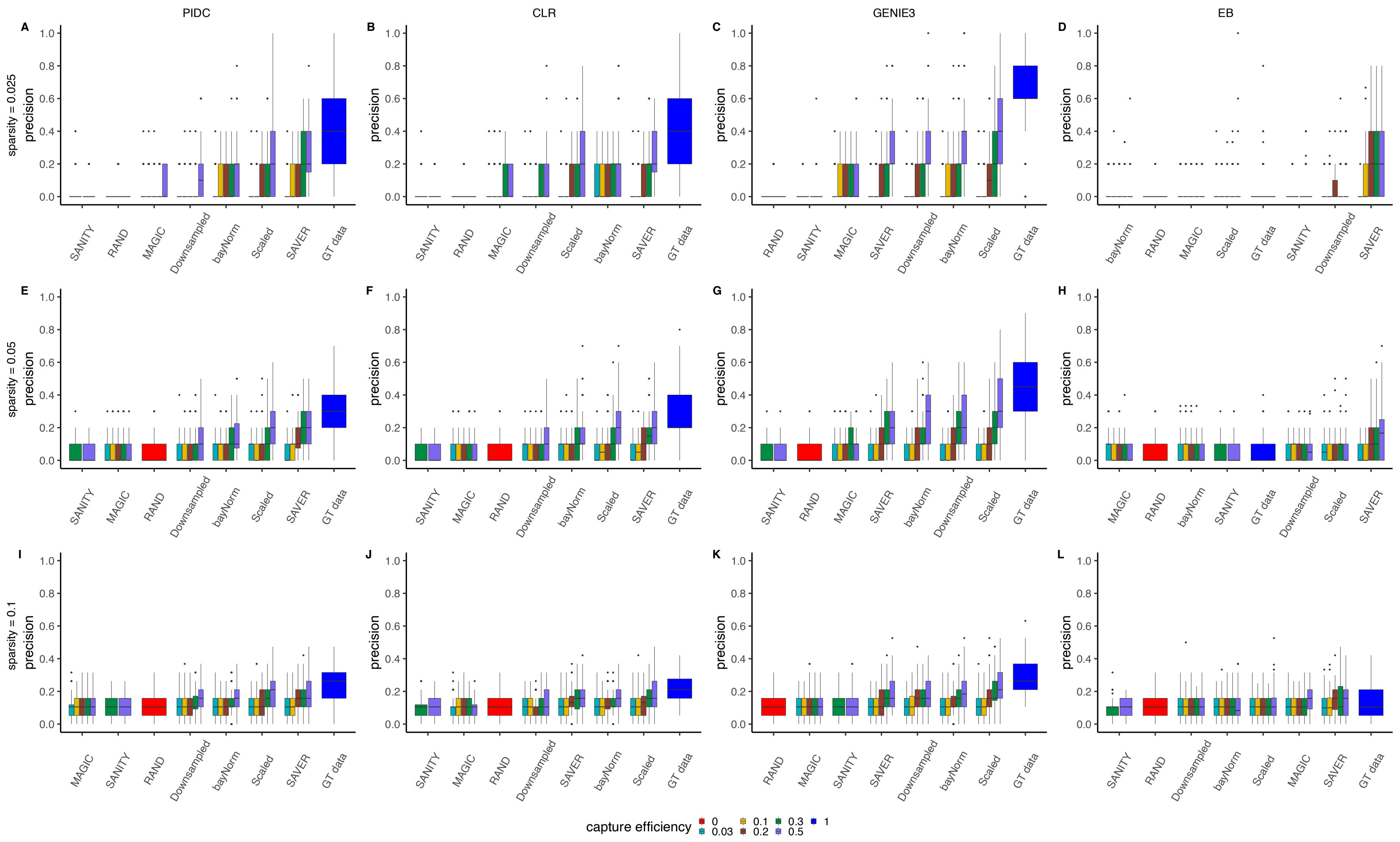

Supplemental Figure 4: Impact of imputation on network inference performance for 100 different simulated 20 gene networks using sparsity = 0.025, 0.05 and 0.1 with **additive regulation** for various captures efficiency. RAND corresponds to precision obtained using random classification and GT data corresponds to precision obtained without downsampling (i.e., capture efficiency is set to 1). Plots (A) to (D) show box-plots of precision scores obtained for different imputation algorithms displayed on x-axes for PIDC, CLR, GENIE3 and Empirical Bayes respectively with sparsity = 0.025. Plots (E) to (H) show box-plots of precision scores obtained for different imputation algorithms displayed on x-axes for PIDC, CLR, GENIE3 and Empirical Bayes respectively with sparsity = 0.05. Plots (I) to (L) show box-plots of precision scores obtained for different imputation algorithms displayed on x-axes for PIDC, CLR, GENIE3 and Empirical Bayes respectively with sparsity = 0.1.

5 Inference precision: 20 gene multiplicative regulation

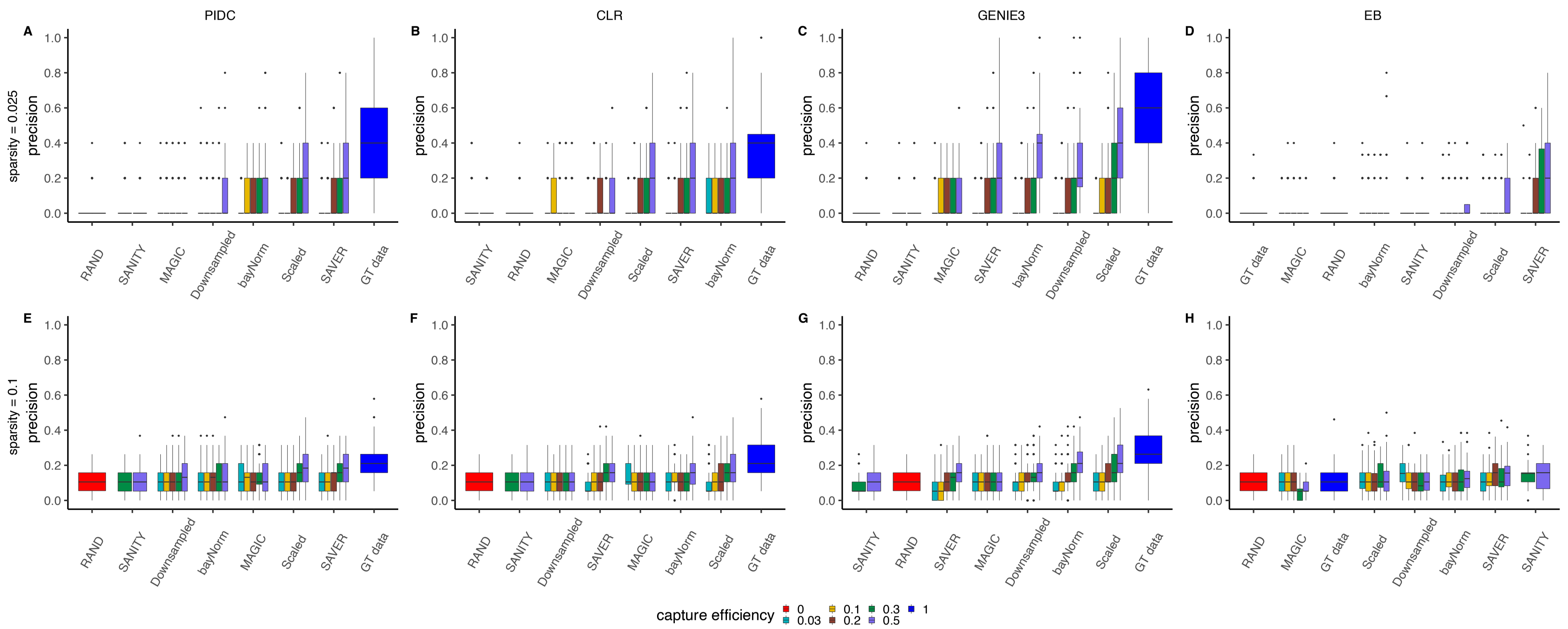

Supplemental Figure 5: Impact of imputation on network inference performance for 100 different simulated 20 gene networks using sparsity = 0.025 and 0.1 with **multiplicative regulation** for various captures efficiency. RAND corresponds to precision obtained using random classification and GT data corresponds to precision obtained without downsampling (i.e., capture efficiency is set to 1). Plots (A) to (D) show box-plots of precision scores obtained for different imputation algorithms displayed on x-axes for PIDC, CLR, GENIE3 and Empirical Bayes respectively with sparsity = 0.025. Plots (E) to (H) show box-plots of precision scores obtained for different imputation algorithms displayed on x-axes for PIDC, CLR, GENIE3 and Empirical Bayes respectively with sparsity = 0.1.

6 Inference precision: 50 gene case, two different sparsity levels

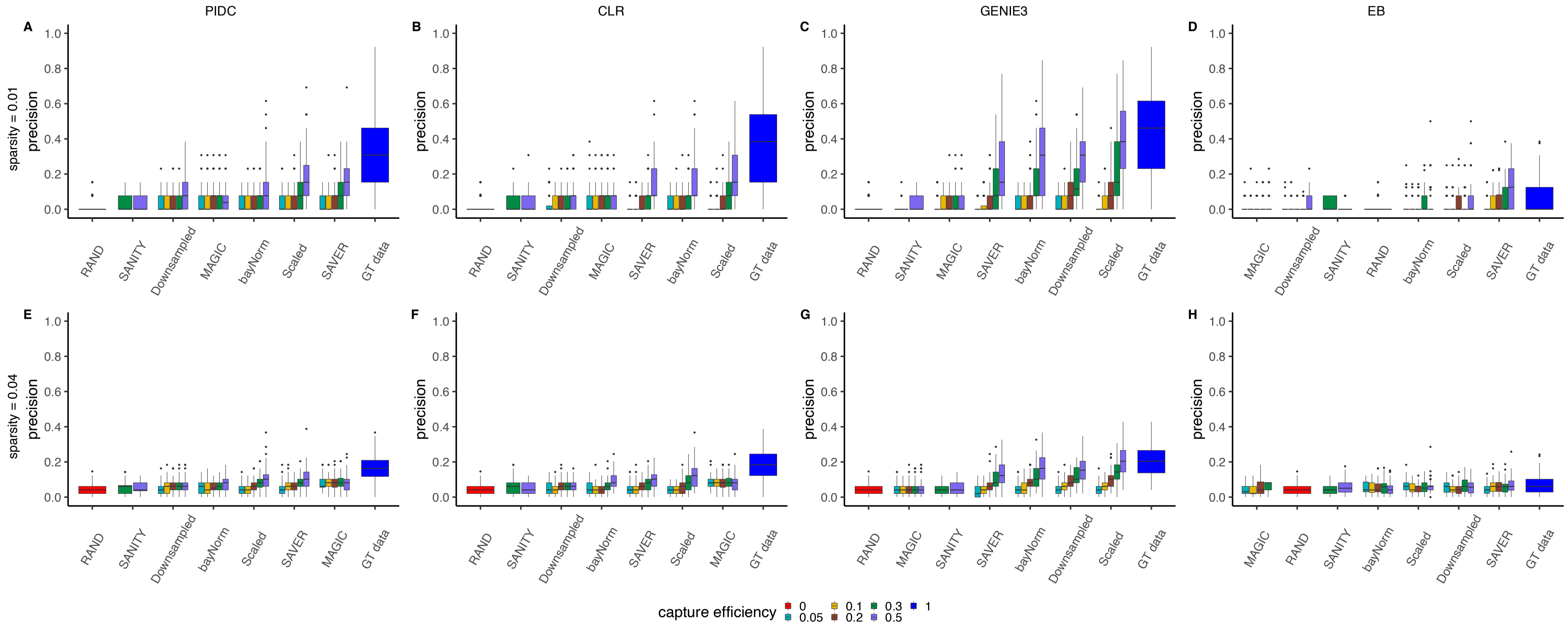

Supplemental Figure 6: Impact of imputation on network inference performance for 100 different simulated 50 gene networks using sparsity = 0.01 and 0.04 with **multiplicative regulation** for various captures efficiency. RAND corresponds to precision obtained using random classification and GT data corresponds to precision obtained without downsampling (i.e., capture efficiency is set to 1). Plots (A) to (D) show box-plots of precision scores obtained for different imputation algorithms displayed on x-axes for PIDC, CLR, GENIE3 and Empirical Bayes respectively with sparsity = 0.01. Plots (E) to (H) show box-plots of precision scores obtained for different imputation algorithms displayed on x-axes for PIDC, CLR, GENIE3 and Empirical Bayes respectively with sparsity = 0.04.

7 Number of combination reactions in both 20 and 50 gene networks

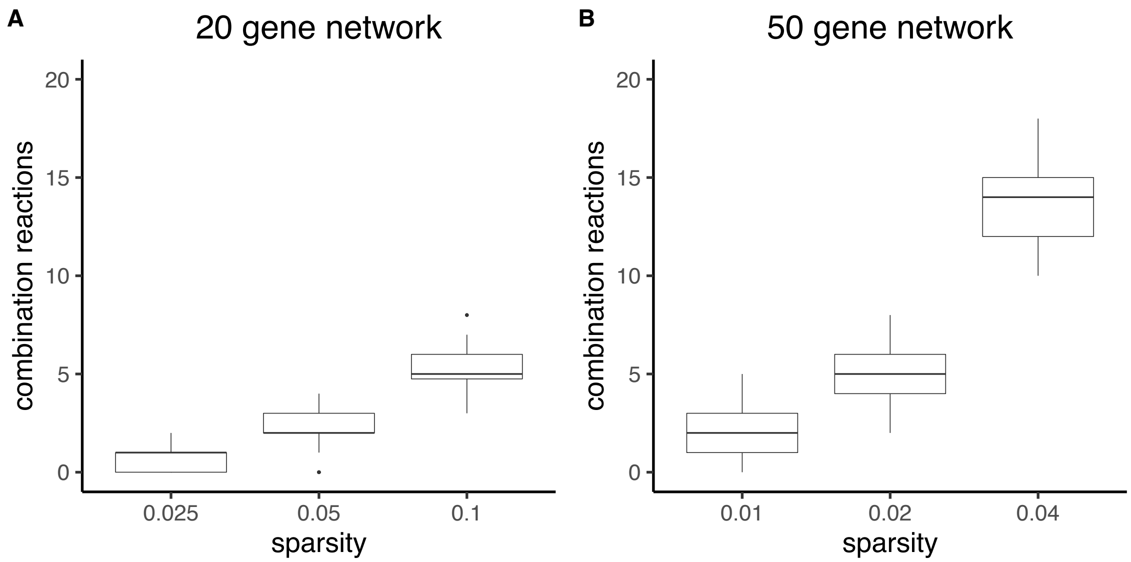

Figure 7: Number of combination reactions in 100 models of 20-genes and 50-genes networks for different sparsity levels. (A) box-plots of the number of combination reactions in 20-genes network for sparsity = 0.025, 0.05 and 0.1. (B) box-plots of the number of combination reactions in 50-genes network for sparsity = 0.01, 0.02 and 0.04.

8 Comparison of 20 gene network with 0.1 sparsity and 50 gene network with 0.02 sparsity

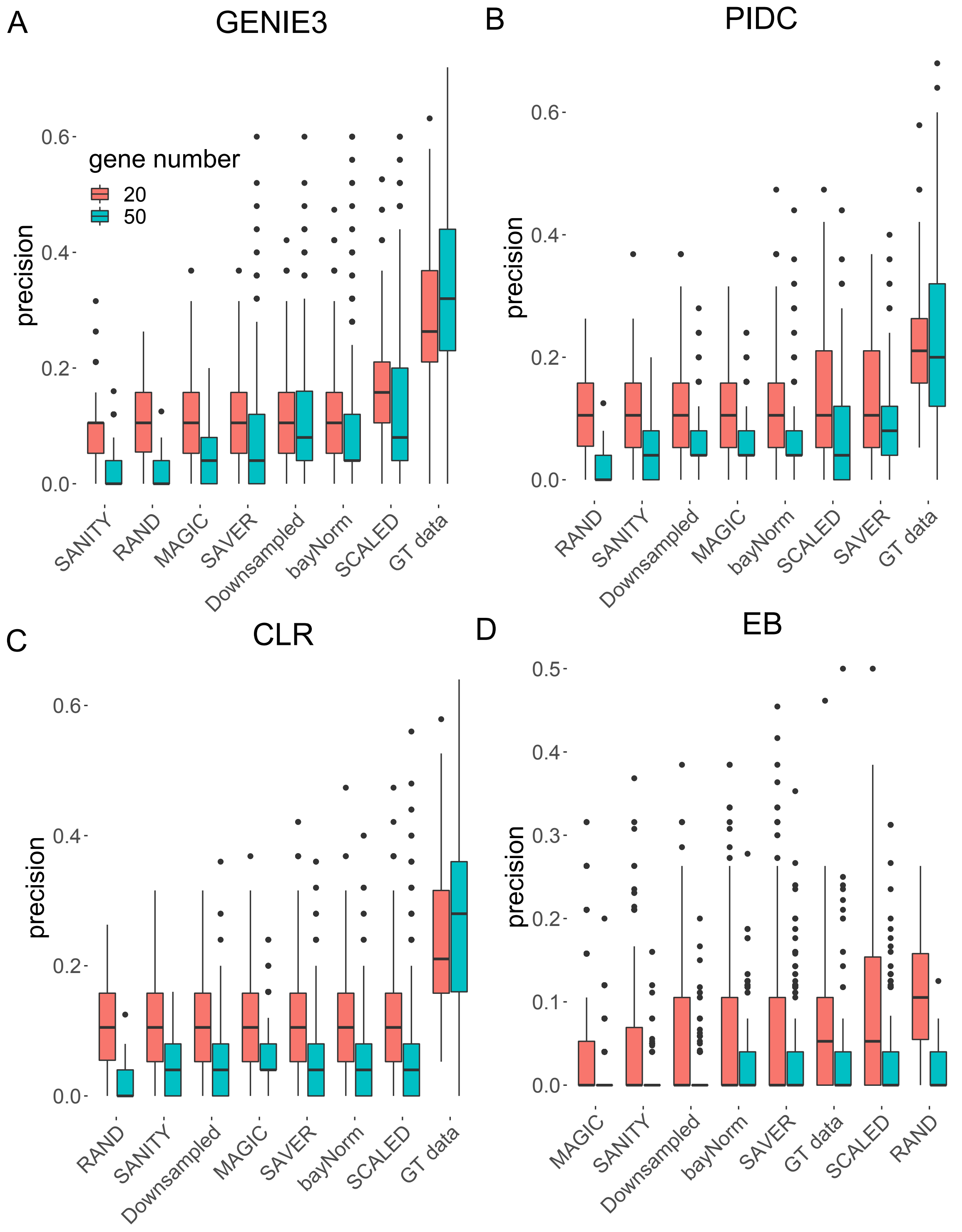

Supplemental Figure 8: Performance of 100 models of 20-genes and 50-genes networks for 0.1 and 0.02 sparsity levels respectively. (A) box-plots of the precision scores for GENIE3. (B) box-plots of the precision scores for PIDC. (C) box-plots of the precision scores for CLR. (D) box-plots of the precision scores for EB.
